# Supplementary material for: The proportion and determinants of COVID-19 infection among medical doctors in Sudan, 2020: A cross-sectional survey
Source: PLoS One. 2022 Nov 4;17(11):e0268037. doi: 10.1371/journal.pone.0268037 (PMC9635736; doi:10.1371/journal.pone.0268037)
Supplement: S1 Table — (DOCX) [file pone.0268037.s001.docx]

**S1 Table: Availability of Infection control measures for COVID-19 at hospitals**

| Availability of Infection control measures for COVID-19 at hospitals | | All | Public hospital | Non-Public (private /military) | X^2^ | p |
| --- | --- | --- | --- | --- | --- | --- |
| PPE | No | 83(23.6%) | 59(71.1%) | 24(28.9%) | 7.8 | 0.00 |
|  | Yes | 269(76.4%) | 147(54.6%) | 122(45.4%) |  |  |
| Hand hygiene | No | 112(31.8%) | 84(75.0%) | 28(25.0%) | 18.3 | 0.00 |
|  | Yes | 240(68.2%) | 122(50.8%) | 118(49.2%) |  |  |
| Triage | I work in a designated hospital for COVID-19 | 40(11.4%) | 28(70.0%) | 12(30.0%) | 2.6 | 0.26 |
|  | No | 93(26.4%) | 55(59.1%) | 38(40.9%) |  |  |
|  | Yes | 219(62.2%) | 123(56.2%) | 96(43.8%) |  |  |
| Temporal isolation | I work in a designated hospital for COVID-19 | 32(9.1%) | 21(65.6%) | 11(34.4%) | 9.2 | 0.01 |
|  | No | 112(31.8%) | 77(68.8%) | 35(31.3%) |  |  |
|  | Yes | 208(59.1%) | 108(51.9%) | 100(48.1%) |  |  |
