# Supplementary material for: The proportion and determinants of COVID-19 infection among medical doctors in Sudan, 2020: A cross-sectional survey
Source: PLoS One. 2022 Nov 4;17(11):e0268037. doi: 10.1371/journal.pone.0268037 (PMC9635736; doi:10.1371/journal.pone.0268037)
Supplement: S1 Questionnaire — (DOCX) [file pone.0268037.s002.docx]

The Proportion and Determinants of COVID-19 Infection among medical doctors in Sudan Hospitals, 2020

This is a voluntary study. Please take the time to decide if you wish to participate or not. If you do wish to participate you will be asked to complete the consent form before starting the survey. Your responses hold a great significance in the survey.

You can stop your participation in the study at any time by simply closing your browser window.

There are no anticipated risks by participating in this study, because we don’t ask for your name, hospital name or your email

Your data will be kept securely for the purpose of the study only

*Required

1. Do you want to participate? *

*Mark only one oval.*

Yes No

1. Age in years? *
2. Gender *

*Mark only one oval.*

Female Male

1. What is your current status? *

*Mark only one oval.*

Consultant/Specialist Registrar

Medical officer House officer

1. Where usually do you work? *

*Mark only one oval.*

ER ICU/HDU/CCU

Wards

Infection Prevention & Control personnel Hospital administration

Other:

1. What is your hospital type? ( Please if you work in two hospital, answer all question based on only one of them) *

*Mark only one oval.*

Public Hospital Private/ NGO Hospital Military Hospital

Other:

1. Where do you work? *

*Mark only one oval.*

Khartoum state Gezira state

North Kordofan state White Nile state

Al Gadarif state Northern state Kassala state Blue Nile state North Darfur state South Darfur state

South Kordofan state River Nile state

Red Sea state Sennar state West Darfur state

Central Darfur state East Darfur state West Kordofan state

1. Have you been working in your hospital after 12/3/2020? *

*Mark only one oval.*

Yes No

1. Have you ever been in contact with a suspected COVID-19 case/s within 2 meters for more than half hour without face mask (you and the patient)? *

*Mark only one oval.*

Yes No

I am not sure

1. Have you ever been in contact with a confirmed COVID-19 case/s within 2 meters for more than half hour without face mask (you and the patient)? *

*Mark only one oval.*

Yes No

I am not sure

1. Have you ever been in physical contact (without glove) with a suspected COVID-19 case/s? *

*Mark only one oval.*

Yes No

I am not sure

1. Have you ever been in physical contact (without glove) with a confirmed COVID-19 case/s? *

*Mark only one oval.*

Yes No

I am not sure

COVID-19 case/s? *

*Mark only one oval.*

Yes No

I am not sure

1. Have you ever been in direct physical contact with secretions or excretion of a confirmed COVID-19 case/s? *

*Mark only one oval.*

Yes No

I am not sure

1. Did you go for home visit for patient/s with respiratory symptoms? *

*Mark only one oval.*

Yes No

I don't know/remember

with suspected or confirmed case/s? *

*Mark only one oval.*

Care provider in charge for the suspected or confirmed case Relative

Colleagues Accidental

Home visit for a patient

I had no contact with a suspected or confirmed COVID-19 case/s Other:

1. If you had a contact with a suspected or confirmed COVID-19 case/s: Where did you come in contact with suspected or confirmed case/s?

*Mark only one oval.*

In the hospital (ER/ward/ ICU etc ...) Ambulance

At home

At public places Residential area (dorm)

I had no contact with a suspected or confirmed COVID-19 case/s Other:

1. Have you been a suspected case yourself (scored 5 according to FMOH COVID-19 guidelines)?

*

*Mark only one oval.*

Yes No

I don't know

1. Have you ever been quarantined after contact with suspected or confirmed case? *

*Mark only one oval.*

Yes No

I had no contact with suspect or confirmed COVID-19 case

1. If you had been quarantined, Who decided that ? *

*Mark only one oval.*

I have never been in quarantine I made this decision by myself

Colleagues advised you to be quarantined Your specialist/ consultant

COVID-19 risk assessment team Medical director

Other:

1. If you have been quarantined, where?

*Mark only one oval.*

I have never been in Quarantine In my hospital

In governmental quarantine At home

In residential area (Dorm)

1. If you have been quarantined, was there any kind of medical follow-up?

*Mark only one oval.*

Yes No

I have never been in Quarantine

1. Have you been tested for COVID-19? *

*Mark only one oval.*

Yes No

1. If you tested, what was the result of the test? *

*Mark only one oval.*

Positive PCR (including Recovered after +ve PCR) Negative PCR

Waiting for the result Not tested

1. If you tested positive, did you work at COVID-19 isolation room two weeks before your tested positive? *

*Mark only one oval.*

Yes No

Not tested/ Negative PCR

1. If you tested positive, from whom did you get the infection? *

*Mark only one oval.*

I do not know exactly From patient/s

From colleague/s

From family member/s or relatives (even if they are medical staff) Not tested/ Negative PCR

1. Is there a COVID-19 Triage in your hospital? (If you had been quarantined or a suspected/confirmed COVID-19 case, we mean two weeks before that) *

*Mark only one oval.*

Yes No

I work in a designated hospital for COVID-19

1. Is there a COVID-19 temporary isolation room in your hospital? (If you had been quarantined or a suspected/confirmed COVID-19, we mean two weeks before that) *

*Mark only one oval.*

Yes No

I work in a designated hospital for COVID-19

1. How often your hospital provides face masks for the staff? (this is a Likert scale question) *

*Mark only one oval.*

1 2 3 4 5

Never Always

1. How often your hospital provides examination gloves for the staff? (this is a Likert scale question) *

*Mark only one oval.*

1 2 3 4 5

Never Always

1. How often your hospital provides hand sanitizer for the staff? (this is a Likert scale question) *

*Mark only one oval.*

1 2 3 4 5

Never Always

1. How often in your hospital there is running water and soap for the staff? (this is a Likert scale question) *

*Mark only one oval.*

1 2 3 4 5

Never Always

1. How often your hospital provides gown for staff in the temporary Isolation room/ Aerosol generating procedures? (this is a Likert scale question) *

*Mark only one oval.*

1 2 3 4 5

Never Always

1. How often your hospital provides goggle/face shield for staff in the temporary Isolation room/ Aerosol generating procedures? (this is a Likert scale question) *

*Mark only one oval.*

1 2 3 4 5

Never Always

1. In your hospital, are FMOH COVID-19 guidelines (hard or soft copies) accessible? *

*Mark only one oval.*

Yes No

I don't know

1. If your hospital had gone for a shut down after encountering a suspected or confirmed COVID-19 case, what was the cause of shut down? *

*Mark only one oval.*

I don't know/ I'm not sure Disinfection process Staff are quarantined

Hospital did not go for shut down Other:

1. In case your hospital had been shut down; how many hours do the disinfection of an area of suspected or confirmed COVID-19 case last usually?
2. Did your hospital arrange any COVID-19 training course/s (even if course/s was/ were in collaboration with MOH or other institutes/ Associations/ NGO)? *

*Mark only one oval.*

Yes No

I don't know

1. Did you attend any COVID-19 training course/s (including an Online course)? *

*Mark only one oval.*

Yes No

1. Thanks for your valuable time, any comments?

This content is neither created nor endorsed by Google.

[Forms](https://www.google.com/forms/about/?utm_source=product&utm_medium=forms_logo&utm_campaign=forms)
